# Experimental assessment of factors mediating the naturalization of a globally invasive tree on sandy coastal plains: a case study from Brazil

**DOI:** 10.1093/aobpla/plw042

**Published:** 2016-08-02

**Authors:** Thalita G. Zimmermann, Antonio C. S. Andrade, David M. Richardson

**Affiliations:** 1 Laboratório De Sementes. Instituto De Pesquisas Jardim Botânico Do Rio De Janeiro. Rua Pacheco Leão, 915, Jardim Botânico, Rio De Janeiro, RJ 22460-030, Brazil; 2 Department of Botany and Zoology, Centre for Invasion Biology, Stellenbosch University, Matieland 7602, South Africa

**Keywords:** Biological invasions, germination, growth, phenotypic integration, phenotypic plasticity, shade, survival, trait, tree invasions, water stress

## Abstract

As all naturalized species are potential invaders, it is important to better understand the determinants of naturalization of alien plants. This study sought to identify traits that enable the alien tree *Casuarina equisetifolia* to overcome barriers to survival and reproductive and to become naturalized on sandy coastal plains. Restinga vegetation in Brazil was used as a model system to conceptualize and quantify key stressors (high temperature, solar radiation, drought and salinity) which can limit the initial establishment of the plants. Experiments were conducted to evaluate the effects of these environmental factors on seed persistence in the soil (field), germination (laboratory), survival, growth, phenotypic plasticity and phenotypic integration (greenhouse). Results show that the expected viability of the seeds in the soil was 50 months. Seeds germinated in a similar way in constant and alternating temperatures (20–40 °C), except at 40 °C. Low light, and water and salt stresses reduced germination, but seeds recovered germination when stress diminished. Young plants did not tolerate water stress (<2 % of soil moisture) or deep shade. Growth was greater in sunny than in shady conditions. Although a low degree of phenotypic plasticity is important in habitats with multiple stress factors, this species exhibited high germination plasticity, although young plants showed low plasticity. The positive effect of phenotypic integration on plastic expression in the shade shows that in stressful environments traits that show greater phenotypic plasticity values may have significant phenotypic correlations with other characters, which is an important factor in the evolutionary ecology of this invasive species. Long-term seed persistence in the soil, broad germination requirements (temperature and light conditions) and the capacity to survive in a wide range of light intensity favours its naturalization. However, *C. equisetifolia* did not tolerate water stress and deep shade, which limit its potential to become naturalized on sandy coastal plain.

## Abstract (precis)

Long-term seed persistence in the soil, broad germination requirements (temperature and light conditions) and the capacity to survive in a wide range of light intensity favours *Casuarina equisetifolia* naturalization. This species exhibited high germination plasticity, although young plants showed low plasticity. The positive effect of phenotypic integration on plastic expression in the shade shows that in stressful environments traits that show greater phenotypic plasticity values may have significant phenotypic correlations with other characters. However, *C. equisetifolia* did not tolerate water stress and deep shade, which limit its potential to become naturalized on sandy coastal plain.

## Introduction

Biological invasions are conceptualized as occurring along an introduction–naturalization–invasion continuum (Blackburn *et al.* 2011; Richardson and Pyšek 2012). As all naturalized species have the potential to become invasive, naturalization is a critical stage of the invasion process (Richardson and Pyšek 2012). For an introduced population to become naturalized, it must overcome biotic and abiotic barriers to survival and reproduction (Blackburn *et al.* 2011). Research on naturalized populations is important for elucidating the ecological factors and species traits that mediate the transition of a population from casual to naturalized, but it is surprising that this phase is rarely explored in studies of invasions (Pyšek *et al.* 2008; Richardson and Pyšek 2012). In general, reproductive traits, such as seed bank longevity, seed germination and seedling survival and growth (Pyšek and Richardson 2007), in addition to high phenotypic plasticity and high phenotypic integration (Pigliucci 2003; Hamilton *et al.* 2005; Richards *et al.* 2006) are considered to be important determinants of invasiveness. However, we know of no studies that evaluate the importance of all these factors together in mediating the transition of a population from casual to naturalize.

High levels of plasticity can increase the average fitness of a species, thereby expressing advantageous phenotypes that facilitate invasion across a wide range of new environments (Richards *et al.* 2006; Funk 2008; Molina-Montenegro *et al.* 2012). Nonetheless, plasticity is not necessarily a crucial factor in invasiveness (Peperkorn *et al.* 2005; Godoy *et al.* 2011; Palacio-López and Gianoli 2011). It seems to be less relevant in habitats that experience the effects of multiple stress factors, where convergence to a low degree of phenotypic plasticity and high canalization may be advantageous (Valladares *et al.* 2007). Considering that the phenotype expressed by plants is the result of the integration of their characters in each environmental condition (Pigliucci 2003), it has been suggested that phenotypic integration (i.e. the pattern and magnitude of functional correlation among different plant traits, Pigliucci 2003), may play a role in constraining phenotypic plasticity (Gianoli 2004; Valladares *et al.* 2007; Gianoli and Palacio-López 2009). An integrated phenotype may have an important advantage in the invasion process because it can respond to environmental variation more efficiently, producing a more adaptive response to the environment than less integrated phenotypes (Schlichting 1989; Gianoli 2004). Consequently, plants with a more integrated phenotype should be less plastic than plants that show lower number of correlations among their traits (Valladares *et al.* 2007; Gianoli and Palacio-López 2009). However, phenotypic plasticity and phenotypic integration can both favour plant fitness (Godoy *et al.* 2012). Further research is thus necessary to elucidate the direction of phenotypic change in invasive species for a better understanding of how ecological traits are inﬂuenced by new environmental conditions (Flores-Moreno *et al.* 2015).

A genus of trees that has been widely planted outside its native range is *Casuarina* (Casuarinaceae) (Potgieter *et al.* 2014*a*). Casuarinas differ from other well-studied invasive trees (e.g. Australian acacias, *Eucalyptus* spp. and *Pinus* spp.; Kueffer *et al.* 2013) in that they invade a distinctive set of habitats (e.g. beach crests, rock coasts, young volcanic ﬂows, riparian ecosystems) and their requirements for successful invasion differ from those of other tree taxa (Morton 1980; Potgieter *et al.* *2014a*, *c*). This genus provides a useful model for understanding how interactions between ecological factors and species traits mediate naturalization and other stages along the introduction–naturalization–invasion continuum (Potgieter *et al.* 2014*a*). *Casuarina equisetifolia* L. is the most widely planted species in the genus and is one of the most invasive alien tree species in the world (Rejmánek and Richardson 2013, Potgieter *et al.* 2014*a*); it invades mainly coastal regions (Wheeler *et al.* 2011). In Brazil, the species was introduced along the entire coast, especially in sandy coastal plains (I3N Brazil 2015). The species is widely naturalized, but it is not yet invasive in this country (Zenni and Ziller 2011; Potgieter *et al.* 2014*a*). Given the large extent of climatically suitable areas for *C. equisetifolia* in Brazil, including many areas with substantial plantings (high propagule pressure), further naturalizations and invasions of this species are likely in the future (Potgieter *et al.* 2014*a*).

Sandy coastal plain ecosystems are characterized by multiple stressful conditions (e.g. high solar radiation, drought, nutrient-poor sandy substrate, high temperatures and salinity, Reinert *et al.* 1997; Hesp and Martínez 2007). These factors have the potential to limit germination, survival and growth of plants (Maun 1994; Scarano 2009). Communities of sandy coastal plains called ‘restinga’ (*sensu* Araújo 1992) occupy 79 % of the Brazilian coast (5.820 km), extending from the Equator to below the Tropic of Capricorn—a distance of ∼3.900 km (67 % in the tropics; Lacerda *et al.* 1993). The restingas occur on sandy soils and have several formations which vary in species composition and vegetation structure, due to varying abiotic conditions (Lacerda *et al.* 1993). Some restingas have a patchy structure and are classified as open scrub vegetation. In many parts of the world, extensive areas of sandy coastal plains are covered by open scrub vegetation that may occur behind the coastal thicket or farther inland (Araújo and Pereira 2002). This vegetation provides a spatial heterogeneity of resources, resulting in two distinct microsites: vegetation patches and open areas (Araújo and Pereira 2002) [**see Supporting Information—Fig. S1]**. Woody species (up to 5 m high) dominate and vines are also common components of the vegetation patches (Araújo and Pereira 2002, Araújo *et al.* 2009). Inside the patches, environmental conditions may be less harsh than in open areas due to higher water supply and lower solar irradiation (Gómez-Aparicio *et al.* 2005). Nevertheless, shade beneath patch canopies can limit plant growth by reducing photosynthesis (Callaway and Walker 1997; Hastwell and Facelli 2003). The two distinct environmental conditions found in the restinga (high irradiance and low water (open area) versus low irradiance and high water (patches) (Matos 2014)) allow for the evaluation of the combined effects of shade and drought in the naturalization process.

The restinga ecosystems are associated with the Brazilian Atlantic Forest domain which is highly degraded; only 11.7 % of the original vegetation remains, which 0.5 % comprises remaining restingas and mangroves (Ribeiro *et al.* 2009).The restinga is highly degraded (Araújo and Pereira 2002; Rocha *et al.* 2007) mainly as a result of vegetation removal for housing development, the collection of plants for sale and the establishment of alien plant species such as *C*. *equisetifolia* (Rocha *et al.* 2007). Despite its high invasive potential and its increasing biological and economic impacts on sandy coastal plains in many parts of the world (Potgieter *et al.* 2014*a*), relatively little is known about the ecophysiological traits that favour *C. equisetifolia* invasiveness. Thus, analysis of seed persistence in the soil, germination behaviour and plant growth performance in response to different environmental factors could allow a better understanding of the factors that make *C. equisetifolia* one of the most widespread invasive trees in coastal regions of the world (Rejmánek and Richardson 2013; Potgieter *et al.* 2014*a*).

The main objective of the study was to identify the sets of traits that enable *C. equisetifolia* to overcome the survival and reproductive barriers (Blackburn *et al.* 2011) and to become naturalized in the restinga. The hypotheses were: (i) *C. equisetifolia* forms a persistent soil seed bank that favours invasion; (ii) given the wide climatic amplitude in its native range (Whistler and Elevitch 2006; Potgieter *et al.* 2014*a*), *C. equisetifolia* seeds can germinate across a broad range of temperatures; (iii) because the species is shade-sensitive and mostly found near water bodies (U.S. National Research Council 1984; Parrotta 1993), drought and shade should reduce its germination, survival and growth; (iv) *C. equisetifolia* should display a low trait plasticity and (v) phenotypic plasticity and phenotypic integration of traits are inversely related in this species (Gianoli 2004; Gianoli and Palacio-López 2009). A better understanding of the traits and the environmental factors that facilitate its naturalization will help to elucidate the magnitude of the invasion debt (*sensu* Rouget *et al.* 2016) for this species in many parts of the world where it has been planted but where invasions have not yet manifested. This study will improve our knowledge about how key stressors (high temperature, solar radiation, drought and salinity) can limit the initial establishment of an alien species and the transition of a population from casual to naturalized. Further, understanding why and under which circumstances species become naturalized may facilitate the prediction of future invasions, determine the best ways to control invasive species, and elucidate the impact of invasive species on native communities (Pyšek and Richardson 2007; Richardson and Pyšek 2012).

## Methods

### Study species

*Casuarina equisetifolia* (Australian pine or coastal she-oak) is an evergreen, fast-growing tree that attains a height of 10–40 m. The species has the largest natural distribution in the genus and is native to the east coast of Australia and Southeast Asia (Parrotta 1993). Reproduction is mainly by seeds (Morton 1980; Apfelbaum *et al.* 1983), but it can also propagate vegetatively (Rentería 2007). Dispersal is mainly by wind (Morton 1980), but also by water (Rentería 2007) and birds (Ferriter *et al.* 2007). The species tolerates saline conditions and low soil fertility (Morton 1980). Symbiotic associations with N-fixing actinomycete in the genus *Frankia* as well as ecto-, endo- and arbuscular mycorrhizal fungi allow *C. equisetifolia* to grow on nutrient-poor substrates (Zhong *et al.* 1995, Diagne *et al.* 2013). It has been planted in coastal regions in many parts of the world, mainly to stabilize dunes and for windbreaks (Morton 1980; Parrotta 1993). *Casuarina equisetifolia* has the capacity to invade open areas in the dunes and replace the native vegetation, threatening biodiversity in coastal regions (Wheeler *et al.* 2011). Further, it produces large amounts of litter, which can limit the establishment of native plants (Hata *et al.* 2010). The species is naturalized in at least 32 countries and it has become invasive in 10 geographical regions, including North America (Florida), Central America, South America, Asia, the Middle East, southern Africa and on many islands (Pacific, Indian Ocean, Atlantic and Caribbean Islands) (Rejmánek and Richardson 2013; Potgieter *et al.* 2014*a*). In Brazil, it was introduced and disseminated mainly after 1950, especially in the restingas of southern, southeastern and northeastern Brazil (I3N Brazil 2015). There are no records of the species being invasive in Brazil, although it is widely naturalized (Zenni and Ziller 2011; Potgieter *et al.* 2014*a*).

### Study area

**Figure 1.**
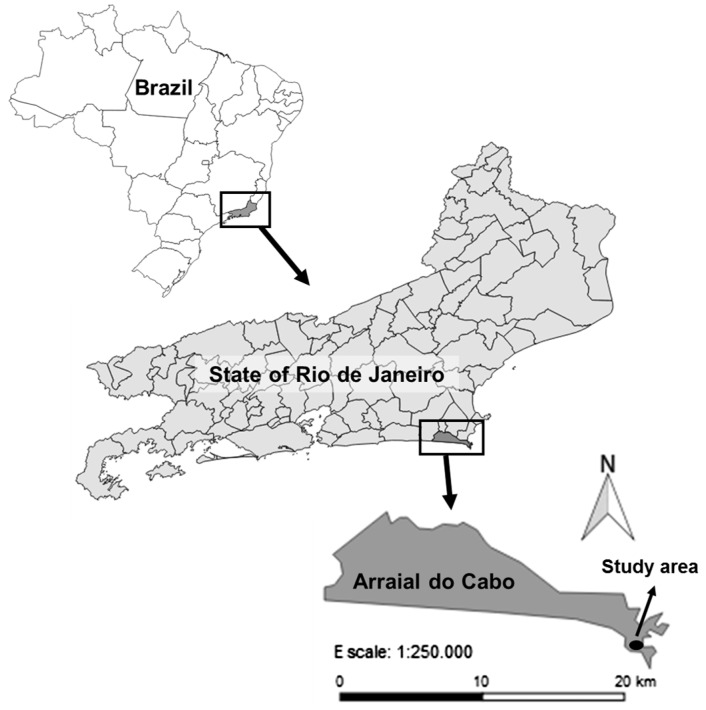
Study area (sea level, 22° 58′S, 42° 01′W) in the restinga of the State Park of Costa do Sol, in the municipality of Arraial do Cabo, State of Rio de Janeiro, Brazil.

The study was conducted in a naturalized population of *C. equisetifolia* (sea level, 22° 58′S, 42° 01′W) in the restinga of the State Park of Costa do Sol, in the municipality of Arraial do Cabo, State of Rio de Janeiro, Brazil (Fig. 1). This is one of the largest *Casuarina* stands (2.2 ha) in the park, and has 0.31 individuals m ^−^ ^2^ (3.048 ind ha ^−^ ^1^), average height of 7.27 ± 3.86 m and diameter at breast height of 5.77 ± 5.18 cm (*n* = 450). In the state of Rio de Janeiro, at least 42 % of restingas are degraded (Rocha *et al.* 2007), but this percentage is now probably substantially higher as disturbance in this ecosystem has increased markedly in recent years (Cosendey *et al.* 2016). The remaining restingas comprise fragments, mostly of small size, with few areas occurring within official Conservation Units (Rocha *et al.* 2007). One of the restingas with the most critical situations in terms of degradation is in the State Park of Costa do Sol (Rocha *et al.* 2007). This restinga is located between the Atlantic Ocean and the Araruama lagoon, the largest hypersaline lagoon in the world. This region is characterized by a hot, semiarid climate, with 800 mm of annual precipitation occurring predominantly during the summer (November to February) (Barbiére 1984). The mean annual temperature is 25 °C, with minimum and maximum temperatures of 12 and 36 °C, respectively (Scarano 2002).

### Seed collection

Approximately 8000 seeds of *C. equisetifolia* were randomly collected from 20 trees, sampled with a minimal distance of 10 m from each other in August 2012. Mature seeds from opened dry dehiscent fruits were dried (18 °C; 18 % relative humidity) for 3–5 days, and hermetically stored in sealed plastic bags at − 20 °C (Bonner 2008).

### Seed longevity in the soil

To evaluate the longevity of *C. equisetifolia* seeds in the soil, the seeds were packed in nylon mesh bags with sterilized (autoclaved at 121 °C for 0.5 h) sandy soil collected in the restinga (open area). Seventy bags (40 seeds per bag) were buried at a depth of 5 cm in the same area as the seeds were collected. Groups of 10 bags were dug up after 1, 3, 6, 9, 12, 18 and 24 months and the viability of the seeds buried in the soil was evaluated in a laboratory by germination tests. To test the effect of the light in germination of buried seeds, germination tests were carried out under light (photoperiod of 8/16 h) and dark conditions. To compare the viability of the seeds (*n* = 2800) buried in the soil with optimal storage conditions, ∼1500 seeds were stored at −18 °C (control group) over the same period that they were buried. Seed germination tests of the control group were carried out under light conditions. Seeds were germinated in Petri dishes (9 cm diameter), lined with two filter paper discs, moistened with 5 mL of distilled water. The germination tests had a randomized design, with five replicates of 40 seeds; the seeds in each bag constituted a replication.

### Seed traits and germination tests

Dry weight and moisture content of the seeds (five replicates of five seeds) were determined according to the low-constant-temperature-oven method (103 °C/17 h; ISTA 1999). Length and width were measured with a digital calliper for 50 samaras (whole winged fruit, including the seed).

Germination tests were carried out to evaluate the effects of temperature, red/far-red light ratio (R:FR), water and salt stresses. The seeds were germinated in Petri dishes (9 cm diameter) lined with two filter paper discs, moistened with 5 mL of distilled water or specific osmotic solutions (sodium chloride (NaCl) or polyethylene glycol 8000 (PEG 8000)). The temperature of the germination chamber was determined by the temperature experiment. Unless light was an intended variable, a regime of 8 h light/16 h darkness was applied (4×20 W white ﬂuorescent lamps; total ﬂux rate of 90 µmol/m^2^/s).

The temperature experiment was represented by constant temperatures of 15, 20, 25, 30, 35 and 40 °C (± 1.0 °C) and by alternating regimes of 25/20, 30/20, 35/20 and 40/20 °C (8/16 h, respectively; the alternating temperature treatment was 8 h in the light at the higher temperature and 16 h in the dark at the lower temperature). In the temperatures of 25, 30 and 30/20 °C the germination was also evaluated in the dark, and the Petri dishes were wrapped in two aluminium foils. The optimal germination temperature was used in light, water and saline stresses experiments.

The light experiment included six R:FR irradiance treatments: 0.0, 0.2, 0.4, 0.6, 0.8 and 1.0. Zero irradiance treatment was produced by wrapping the Petri dishes in two aluminium foils. The greatest R:FR treatment (1.0) was obtained by leaving the Petri dishes free of filters. Spectrum was provided by two ﬂuorescent 22 W white lamps and one incandescent 15 W lamps, totalling 1.0 R:FR, which is close to the 1.19 R:FR of full sunlight (Smith 2000). The four remaining R:FR irradiance treatments were achieved by wrapping the Petri dishes with different colours of LEE filters. The R:FR irradiance was measured with sensors SKR 110 and SKP 215, coupled to SpectroSense (Skye Instruments Inc.).

The effect of water and salt stresses in the germination was tested with PEG 8000 and NaCl solutions, respectively. The osmotic potentials used were: 0.0, −0.25, −0.5, −0.75, −1.0, −1.25 and −1.5 MPa. These different potentials were found in the restinga (Martins *et al.* 2012). PEG 8000 and NaCl solutions were prepared according to Villela and Beckert (2001) and Salisbury and Ross (1992), respectively. To minimize water potential variation, seeds were transferred to a new Petri dish with the solution every 7 days. After 30 days, in a recovery treatment, the ungerminated seeds from PEG 8000 and NaCl solutions were washed with distilled water. The seeds were then transferred to Petri dishes with distilled water to evaluate the germination potential.

In all experiments, the positions of Petri dishes inside germination chambers were randomly changed every day. A seed was considered to have germinated when its radicle emerged to a length of 1 mm. Germination was recorded daily for 30 days, and germinated seeds were removed from Petri dishes. In the light experiment, the germination was evaluated in a dark and closed room, with a green safelight. Five replicates of 40 seeds were used in all experiments. Seeds that did not germinate were subjected to the application of pressure with tweezers, and were either empty or had been colonized by fungi.

### Survival and growth

To minimize genetic variation, all seeds used in this experiment came from a single tree, so the seedlings were half-siblings. Seeds were germinated in germination chambers (30 °C; 8 h photoperiod) and after 2 months, seedlings were transplanted to individual plastic bags (2L) and transferred to the greenhouse of the Rio de Janeiro Botanic Garden. Soil substrate consisted of 1:1:1 volume homogenized mixture of soil of the area with *C. equisetifolia* invasion, sand collected inside the patches and bare sand. This mixture was used to provide a substrate with macro and micronutrients found in the restinga.

After 4 months, the height and stem diameter of the young plants of *C. equisetifolia* were measured. These plants were submitted to a factorial experiment to simulate the light intensity and water availability found in three microsites of the restinga (inside vegetation patches, edge and open area) and in the *C. equisetifolia* stands. This experiment had eight treatments, with four light levels and two watering regimes. The plants were separated in eight groups and there were no significant differences in initial height of the individuals between groups (*P* < 0.05). Distinct conditions of light were established with shade cages of wood (1 m×1 m×1 m), covered with cloth layers of different colours and thicknesses. The photosynthetic photon-flux density (PPFD%) and R:FR (mol mol ^−^ ^1^) inside each shade cage were: ∼2 %, 0.29 mol mol ^−^ ^1^ (inside vegetation patches); ∼15 %, 0.48 mol mol ^−^ ^1^ (edge); ∼70 %, 1.05 mol mol ^−^ ^1^ (*C. equisetifolia* stand) and ∼100 %, 1.12 mol mol ^−^ ^1^ (open area). At each light intensity, half of the young plants were grown under high water (>10 % of soil water content) and other half at low water conditions (<2 % of soil water content). Soil water content was monitored weekly from four soil samples per treatment, and was determined by gravimetric method (24 h/103 °C). The soil was irrigated once or twice a week by applying 30 (2 %, low water) to 150 ml (100 %, high water) of water.

The values of PPFD%, R:FR and watering regimes inside patches, edge and open area in the restinga were obtained by Matos (2014). Data of PPFD% and R:FR of *C. equisetifolia* stands were measured at 20 random points (68.5 ± 11.2 % PPFD%, 1.05 ± 0.10 µmol m ^−^ ^2^ s ^−^ ^1^). The values of PPFD% were calculated taking as reference the mean full sunlight (100 % PPFD = 2305.3 µmol m ^−^ ^2^ s ^−^ ^1^). All measurements were made at midday, on sunny cloud-free days, with a radiometer SKR-100 linked to a SpectroSense 2 SKL 904 (Skye Instruments, Llandrindod Wells, UK). To minimize experimental error due to light variability inside the shade cages, positions of the young plants were rotated once a week. For survival analysis, 15 individuals per treatment were monitored weekly, for 16 weeks. Plants that lost all their aerial structure and did not have any photosynthetic active leaf were recorded as dead.

At the end of the experiment, samples of all young plants that survived were harvested to measure stem length, main root length and collar diameter. Thereafter, they were separated into leaves stems and roots, and each fraction was dried (80 °C/48 h) and weighted. Total dry mass (TDM), Leaf mass fraction (LMF = leaf dry mass/plant dry mass), stem mass fraction (SMF = stem dry mass/plant dry mass), root mass fraction (RMF = root dry mass/plant dry mass), shoot: root ratio (RS = shoot dry mass/root dry mass), slenderness index (SI = stem height/collar diameter), specific stem length (SSL = stem length/stem dry mass), specific root length (SRL = root length/root dry mass), total leaf mass (TLM), total leaf area (TLA), specific leaf area (SLA = leaf area/total leaf mass) and leaf area ratio (LAR = leaf area/total plant dry mass). Leaf area and SLA were calculated following the protocol proposed by Gómez-Aparicio *et al.* (2006) for pines needles. Relative growth rates were calculated for total biomass (RGRb) and total leaf area (RGRa) using the pairing method (Evans 1972). RGR was calculated as RGR = (ln*x*_2_−ln*x*_1_)/(*t*_2_−*t*_1_), where *x_1_*is the trait measured in time 1 (*t*_1_) and *x*_2_ is the trait measured in time 2 (*t_2_*).

### Phenotypic plasticity and phenotypic integration

Phenotypic plasticity in response to light for each trait was calculated as the relative distance plasticity index (RDPI = ∑(dij → i′j′/(xi′j′ + xij))/*n*), where *n* is the total number of distances, and *j* and *j*′ are two individuals belonging to different treatments (*i* and *i*′). This index ranges from 0 (no plasticity) to 1 (maximal plasticity). Overall RDPI was calculated by summing all relative distances obtained and dividing by the total number of distances (Valladares *et al.* 2006). It was not possible to calculate RDPI in relation to water regime because almost all young plants died under low water conditions.

Phenotypic integration was estimated as the number of significant correlations (*P* < 0.05; Spearman’s rank correlation coefficient) with the other traits (pairwise comparison) for 15 % of light (shady condition) and 100 % of light (sunny condition) (Gianoli and Palacio-López 2009). Phenotypic integration index in each light condition was calculated based on the variance of the eigenvalues of the correlation matrix between phenotypic traits (Wagner 1984).

### Data analysis

In the experiments to determine seed longevity in the soil and the effect of temperature, PEG 8000 and NaCl solutions, germination was evaluated by germination percentage and germination rate (*v* = Σni/(Σni·ti)); where ‘ni’ is the number of seeds germinated per day and ‘ti’ is the incubation time (days) (Labouriau and Pacheco 1978). In the light experiment only the final germination percentage was evaluated.

The longevity of *C. equisetifolia* seeds in the soil and cold conditions was analysed through germination percentage and germination rate parameters by linear regression. An analysis of covariance (ANCOVA) was used to compare the slopes of regression lines between the two storage conditions of the seeds (cold storage X soil storage) and the effect of the light conditions on germination of the buried seeds in the soil (light X dark). The ANCOVA was used with germination percentage and germination rate as dependent variables, storage and light conditions as factors and storage time (1, 3,…, 24 months) as covariate. The interaction between the conditions and time in the germination process was evaluated. Homogeneity of slopes was confirmed before conducting each ANCOVA. The differences in ANCOVA were in relation to the inclination.

The recovery germination percentage in the PEG 8000 and NaCl solutions was calculated by adding the germination values of each iso-osmotic solution and their respective germination value after transferal to distilled water. In the experiments of temperature, PEG 8000 and NaCl solutions data were analysed for normality using the Kolmogorov–Smirnov test and for homogeneity of variance using Levene’s test. For data that did not show normality and/or variance homogeneity, germination percentage was arcsine √ transformed and germination rate transformed to log(*x *+ 1) (Zar 1999). Germination percentage and germination rate were tested in a factorial ANOVA, followed by a *post* *hoc* Tukey’s test (*P* < 0.05). In the experiment of light the relationship between germination percentage (y) and R:FR (*x*) was determined using a logistic function (Pearson *et al.* 2003) and described by the following equation: *y* = *a*/{1 + exp [−((*x*−*x*_0_)/*b)*]}, where *a* is a coefficient describing the maximum germination percentage, *x*_0_ is a coefficient estimating the R:FR at 50 % of maximum germination and *b* is a coefficient of the slope of the germination response calculated from estimates of R:FR.

For survival analysis the Kaplan–Meier product limit method was used to estimate the survival function, and the log-rank test was used to assess for significant differences in survival curves among treatments. Cox regression was used to evaluate the effects of light, water and their interactions on probability of the death of young plants.

Growth analyses were performed only in treatments of 15, 70 and 100 % of light under high water conditions due to high mortality rates under low water conditions and in deep shade (2 %). To test the effect of light for all morphological and biomass allocation traits together Multivariate analysis of variance (MANOVA) was used. Traits that showed a significant effect in the MANOVA results were tested separately by one-way ANOVA, followed by a *post hoc* Tukey’s test (*P* < 0.05). Before the analyses, normality of the data was tested by Shapiro–Wilk’s *W* test and homoscedasticity by Levene’s test. To check the homogeneity of covariance matrices Box M test and the Bartlet’s test was used to check for sphericity. Where necessary, data were ln-transformed to correct for deviations from these assumptions. Differences in RGR were submitted to a one-way ANOVA, using Tukey’s *post hoc* test (*P* < 0.05). To minimize the influence of outliers and reduce the within-harvest-variation, prior to growth analysis data were trimmed by the removing the smallest and the largest plant from each treatment (Barnett and Lewis 1978).

Regression analysis was used to determine whether phenotypic plasticity in response to light (dependent variable) and phenotypic integration of traits in shady and sunny conditions (independent variable) are inversely related in *C. equisetifolia*. Values of RDPI were log-transformed before analysis [log(*x* + 1)]. To test the statistical significance between phenotypic integration indices across light conditions, 95 % confidence intervals for the overall R obtained in each environment were calculated by bootstrapping 1000 times (García-Verdugo *et al.* 2009).

Survival analysis was done using the ‘survival’ package (Therneau 2015) and phenotypic integration index and percentage of maximum possible integration were calculated using the ‘PHENIX’ package (Torices and Muñoz-Pajares 2015) in R version 3.0.3 (R Development Core Team 2014). The other analyses were done in Statistica (version 7.0, Statsoft Inc., Tulsa, OK). Graphical display was performed with R and Origin (version 8.0, OriginLab, MA, Cary, NC).

## Results

### Seed longevity in the soil

**Figure 2.**
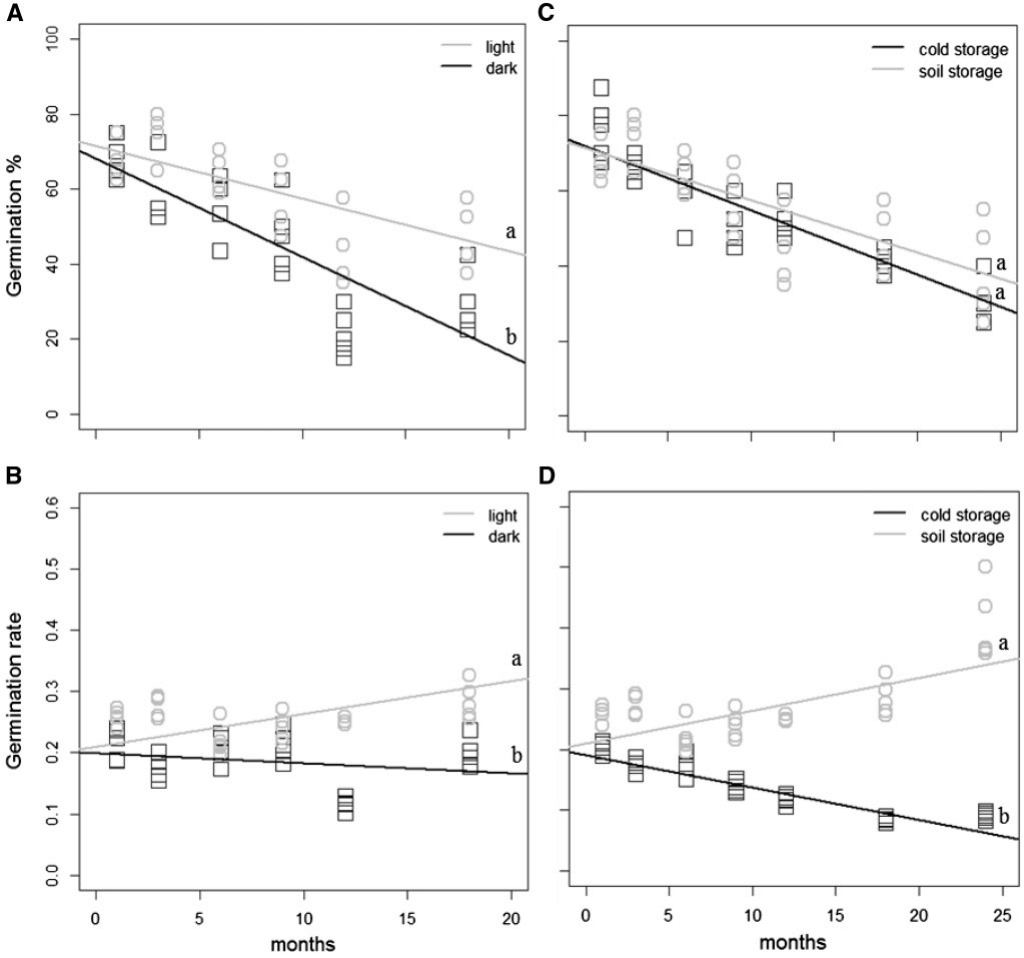
Relationships between storage period (months) and germination percentage (A and C) or germination rate (B and D) for *Casuarina equisetifolia* L seeds. (A and B) Germination percentage and rate of the seeds buried in the soil under light (photoperiod of 8/16 h; grey circles) and under dark (black squares) conditions; (C and D) germination percentage and rate of the seeds stored in cold conditions (black squares) and buried in the soil (grey circles) under light conditions. Data points were fitted with a linear regression function. Germination percentage: light = soil storage (*y* = −1.50*x* + 72.11; *R*^2 ^=^ ^0.52; *P* < 0.001); dark (*y* = −2.63*x* + 68.17; *R*^2 ^=^ ^0.67; *P* < 0.001); cold storage (*y* = −1.71*x* + 71.87; *R*^2 ^=^ ^0.77; *P* < 0.001). Germination rate: light = soil storage (*y* = 0.0009*x* + 0.25; *R*^2 ^=^ ^0.005; *P* = 0.29); dark (*y* = −0.002*x* + 0.20; *R*^2 ^=^ ^0.03; *P* = 0.20) and cold storage (*y* = −0.005*x* + 0.20; *R*^2 ^=^ ^0.84; *P* < 0.001). Different letters denote significant differences between the curves with ANCOVA (*P* < 0.05) [**see Supporting Information—Table S2**].

*Casuarina equisetifolia* seeds remained viable in the soil for at least 24 months, germinated under light and under dark (Fig. 2A) and had a predicted seed viability of 51.1 months (*y* = 71.53−1.40*x*). The interaction between storage condition and storage time was significant for germination rate (ANCOVA, *F* = 90.19, *P* < 0.001) but not for germination percentage (ANCOVA, *F* = 1.18, *P* = 0.28). There were no significant interactions between light conditions and storage time for germination percentage (ANCOVA, *F* = 6.72, *P *= 0.12) and rate (ANCOVA, *F* = 2.89, *P *= 0.09) **[see Supporting Information—Table S2]**.

Germination percentage decreased over time (*R*^2 ^=^ ^0.55, *P* < 0.001), but germination rate was not affected by the storage time (*R*^2 ^<^ ^0.001, *P* = 0.95). In relation to the two storage conditions, there was no significant difference in germination percentage (ANCOVA, *F* = 1.98, *P* = 0.16; Fig. 2C). Nevertheless, germination rate was significantly higher in seeds stored in the soil than at −18 °C (ANCOVA, *F* = 104.34, *P* < 0.001; Fig. 2D). For seeds buried in the soil, germination percentage and rate were significantly higher under light than under dark conditions (ANCOVA, *F* = 25.62, *P* < 0.001; *F* = 55.08, *P* < 0.001, respectively; Fig. 2A and B) **[see Supporting Information—Table S2]**.

### Seed traits and germination tests

**Figure 3.**
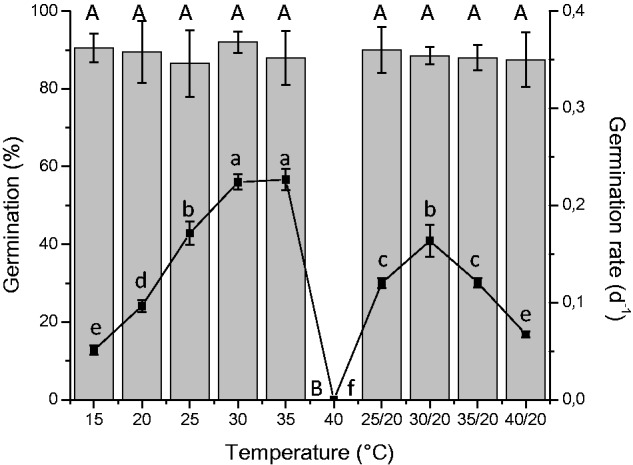
Seed germination (mean ± SD) of *Casuarina equisetifolia* at constant and alternating temperatures. Different upper case letters indicate significant differences in germination percentage (bars, left *y*-axis) and different lower case letters indicate significant differences between germination rate (line, right *y*-axis). ANOVA, *post hoc* Tukey’s test (*P* < 0.05).

*Casuarina equisetifolia* samaras had a dry weight of 0.75 ± 0.12 mg, moisture content of 10.8 ± 1.7 %, length of 5.9 ± 0.5 mm and width of 3.1 ± 0.3 mm. Under light, there were no significant differences in relation to constant and alternating temperature regimes, except for the constant temperature of 40 °C, which completely inhibited germination. The conditions that promoted the highest values of germination rates were 30 and 35 °C (Fig. 3). Thus, 30 °C was chosen as optimal germination temperature for *C. equisetifolia* and was used in the other germination experiments. Germination percentage at 25 and 30 °C was significantly reduced under dark compared to the light conditions (Table 1). Nevertheless, an alternating temperature of 30/20 °C did not have significant differences between the two regimes of light. The absence of luminosity reduced germination rate at all temperatures.

**Table 1:** Light and temperature effects on seed germination (mean ± SD) of *Casuarina equisetifolia.*

Temperature (°C)	**Germination (%)**		**Germination rate (d^−1^)**	
	Light	Dark	Light	Dark
25	86.5±8.6 a	16.5±6.5 b	17.1±1.2 a	12.7±0.9 b
30	92.0±2.7 a	54.0±5.5 b	22.4±0.8 a	11.6±0.3 b
30/20	88.5±2.2 a	92.5±4.0 a	16.4±1.7 a	14.1±0.6 b

Letters denote significant differences between the treatments (Student’s *t*-test, *P* < 0.05).

**Figure 4.**
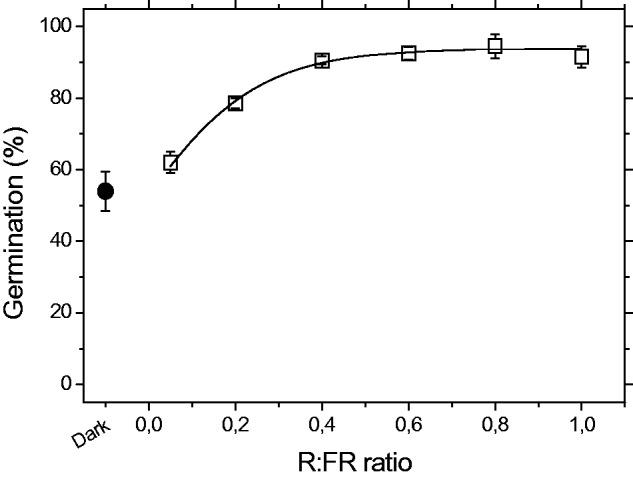
Effect of red/far-red light (R:FR) ratios on mean final germination percentage (±SD) of *Casuarina equisetifolia* L. Data points were fitted with a sigmoidal regression function (solid line; *R*^2 ^=^ ^0.983; *P* < 0.05).

*Casuarina equisetifolia* seeds responded significantly to the treatments involving exposure to the various R:FR ratios (Fig. 4). Seeds were considered neutral photoblastic and showed higher germination percentages in light than in dark conditions. Seed germination increased slightly up to the higher R:FR, as indicated by the good fit to the data (*R*^2 ^=^ ^0.981; *P* < 0.01) provided by the regression analysis. Germination was also sensitive to water and salt stresses, but the decrease in germination percentage and rate was higher in PEG 8000 than in NaCl solution (Table 2). Significant decreases in germination percentages were observed from the water and salt potential of −0.5 and −0.75 MPa, respectively. In both osmotic solutions germination was null from −1.0 MPa. Germination rate dropped as water and salt potentials decreased. After the seeds were transferred to distilled water (recovery treatment), total germination percentage in all treatments showed no significant differences from the control (Table 2).

**Table 2:** Mean (± SD) germination percentage and germination rate of *Casuarina equisetifolia* seeds in response to osmotic (sodium chloride—NaCl) and water (polyethylene glycol 8000—PEG 8000) potential and recovery treatments.

Treatment	Potentials (MPa)	Germination (%)	Germination rate (10^−2^)	Recovery germination (%)
**NaCl**	0.00	91.5±4.2 a	20.3±1.8 a	91.5±4.2 ns
	−0.25	88.5±4.5 a	13.7±2.6 b	88.5±4.5
	−0.50	86.5±1.4 a	8.0±0.3 c	92.0±4.1
	−0.75	43.5±5.2 b	6.3±0.3 c	86.0±2.8
	−1.00	0 c	0 d	84.0±5.8
	−1.25	0 c	0 d	94.0±2.9
	−1.50	0 c	0 d	85.0±7.3
**PEG 8000**	0.00	91.5±4.2 a	20.3±1.8 a	91.5±4.2 ns
	−0.25	84.5±7.8 a	9.9±1.2 b	84.5±7.8
	−0.50	57.0±6.9 b	5.3±0.2 c	93.5±5.8
	−0.75	3.0±1.1 c	4.3±0.3 c	90.0±5.0
	−1.00	0 c	0 d	92.5±4.7
	−1.25	0 c	0 d	93.0±2.1
	−1.50	0 c	0 d	90.5±4.1

The letter codes indicate homogeneous groups among treatments, ns, not significant (Tukey’s test, *P* < 0.05).

### Survival and growth

**Figure 5.**
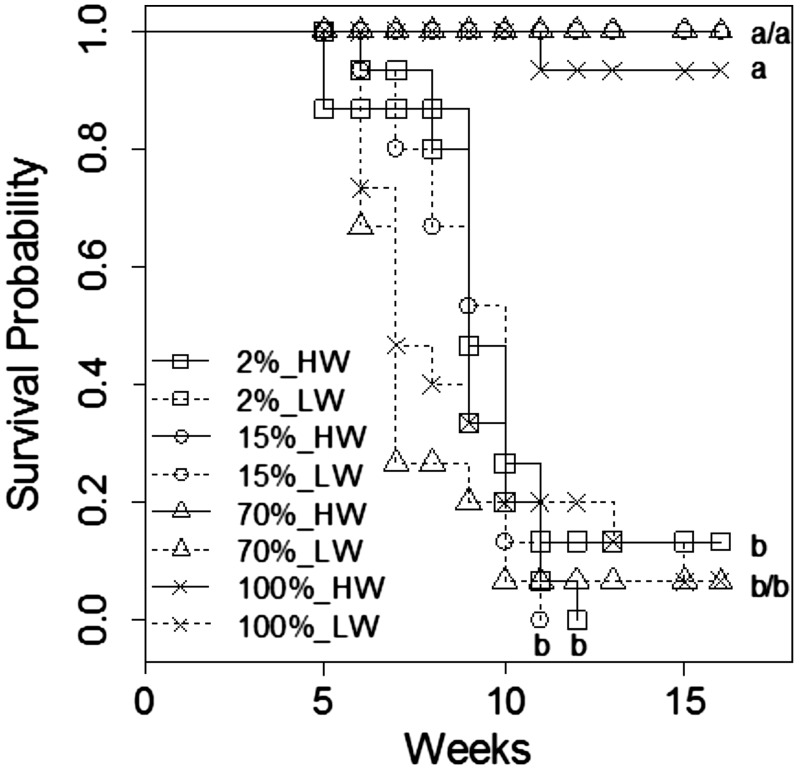
Survival curves of young plants of *Casuarina equisetifolia* (*n* = 15) under combined effects of light (2, 15, 70 and 100 %) and water regimes (HW—high water, LW—low water) over 16 weeks. Survival analysis was performed with the Kaplan–Meier product limit method. The letter codes indicate homogeneous groups (log-rank test, *P* < 0.05).

Survival rates of the young plants had a different response to the combined effect of light and water stress (Fig. 5). Survival was improved under high water conditions **[see ****Supporting Information—Fig. S3A****]** and the probability of death was ∼46 times higher under low water than under high water conditions (Hazard Ratio = 45.97, Wald’s *P* value < 0.001). Similarly, 2 % light conditions had a negative effect on survival rates. Deep shade increased the risk of mortality almost 4 times (Hazard Ratio = 3.70, Wald’s *P* value = 0.03). There were no significant differences between survival rates at 15, 70 and 100 % of light **[see** **Supporting Information—Fig. S3B****]**. Under high water regime, survival was significantly lower at 2 % light, while there were no significant differences in survival between the light regimes under dry conditions (Fig. 5). The interaction between light and water was significant (Wald’s *P* value = 0.008) because the effect of drought was higher under high light (70 and 100 % of light) than under low light (2 and 15 % of light) **[see** **Supporting Information—Fig. S3C and D****]**.

Light intensity had a significant effect in all morphological and biomass allocation traits that were measured (Table 3). Shade conditions (15 % of light) led to significantly lower values of relative growth rates in total biomass and total leaf area and root mass fraction. At high light (70 and 100 % of light), leaf and shoot mass fraction, shoot: root fraction and slenderness index, were significantly lower than under shade. Young plants growing under shady conditions had higher values of specific leaf area and leaf area ratio than plants under sunny conditions. The only trait that differed significantly between 70 and 100 % light was specific stem length (Table 3).

**Table 3:** Mean ± SD, *F* and *P* values (one-way ANOVA) on data for 12 morphological and *biomass allocation* traits of young *plants of* *Casuarina equisetifolia* in response to three light levels (15, 70 and 100 % of photosynthetic photon-flux density) after 16 weeks.

Traits	**15** **%**	**70** **%**	**100** **%**	*F*	*P*
RGRb	0.02±0.00 b	0.04±0.00 a	0.04±0.00 a	168.88	<0.01
RGRa	0.02±0.02 b	0.03±0.00 a	0.03±0.00 a	24.85	<0.001
LMF	0.58±0.02 a	0.48±0.02 b	0.48±0.04 b	62.15	<0.001
SMF	0.20±0.01 a	0.14±0.01 b	0.16±0.02 b	35.27	<0.001
RMF	0.22±0.02 b	0.38±0.03 a	0.36±0.05 a	76.68	<0.001
SR	0.88±0.11 a	0.39±0.05 b	0.45±0.12 b	95.40	<0.001
SI	170.85±19.07 a	69.01±6.77 b	63.02±5.44 b	385.02	<0.01
SSL	129.09±21.33 a	33.97±3.96 b	28.95±3.12 c	480.98	<0.01
SRL	99.78±20.46 a	13.21±3.46 b	13.67±3.39 b	28.79	<0.001
SLA	227.65±20.93 a	116.62±20.96 b	113.07±15.08 b	153.26	<0.01
LAR	132.77±14.43 a	56.56±11.57 b	54.23±7.88 b	195.87	<0.01

The traits shown are relative growth rate in total biomass (RGRb) and total leaf area (RGRa), leaf mass fraction (LMF), stem mass fraction (SMF), root mass fraction (RMF), shoot: root ratio (SR), slenderness index (SI), specific stem length (SSL), specific root length (SRL), specific leaf area (SLA) and leaf area ratio (LAR). the letter codes indicate homogeneous groups among treatments for light intensities (Tukey’s test, *P*< 0.05).

### Phenotypic plasticity and phenotypic integration

**Figure 6.**
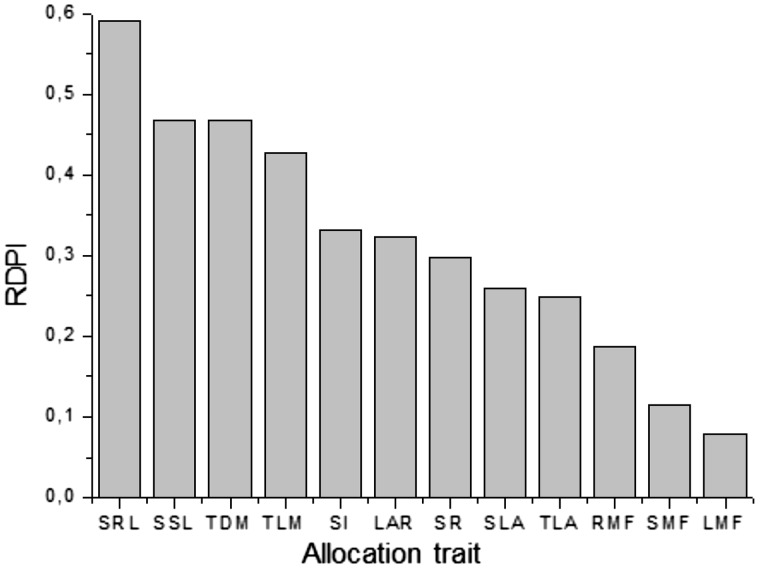
Relative Distance Plasticity Index (RDPI) for 12 allocation traits of young plants of *Casuarina equisetifolia* in response to three levels of light (15, 70 and 100 % of photosynthetic photon-flux density) after 16 weeks. The traits shown are specific root length (SRL), specific stem length (SSL), total dry mass (TDM), total leaf mass (TLM), slenderness index (SI), leaf area ratio (LAR), shoot: root ratio (SR), specific leaf area (SLA), total leaf area (TLA), root mass fraction (RMF), stem mass fraction (SMF) and leaf mass fraction (LMF). The RDPI values range from 0 (no plasticity) to 1 (maximal plasticity).

The overall value of RDPI was 0.32. Phenotypic plasticity in response to light changed in relation to the trait. The value of RDPI ranged between 0.08 (LMF) until 0.59 (SRL) (Fig. 6). Trait plasticity could be ranked as: SRL > SSL >  TDM > TLM >  SI > LAR > SR > SLA > TLA > RMF >  SMF > LMF.

**Figure 7.**
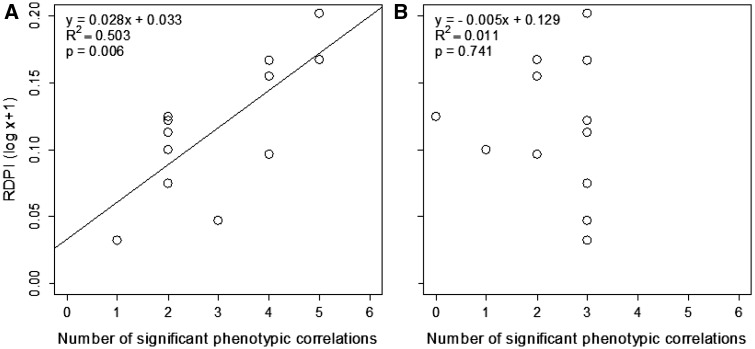
Regression analysis between Mean Relative distance plasticity index (RDPI) in response to light and Phenotypic Integration (PI) in response to shady (15 % of light; A) and sunny conditions (100 % of light; B) among 12 morphological traits of young plants of *Casuarina equisetifolia*. Each point in the regression analysis corresponds to a single trait.

The phenotypic integration index and 95 % confidence intervals overlap between light conditions (15 % =  2.78 ± 1.97; 70 % = 2.53 ± 1.83; 100 % = 2.20 ± 1.40). The magnitude of individual correlations between the traits changed from one environment to another **[see Supporting Information—Table S4]**. Phenotypic plasticity was positively associated with phenotypic integration under shade (*R*^2 ^=^ ^0.51, *P* = 0.006) (Fig. 7). Under sunny conditions, plasticity and integration of the traits showed no significant relationship (*R*^2 ^=^ ^0.011, *P* = 0.74).

## Discussion

Although *Casuarina equisetifolia* invades mainly coastal regions (Wheeler *et al.* 2011), abiotic conditions in the restingas can limit the naturalization of introduced populations of this species. High temperatures prevent seed germination and low light affects the survival and growth of young plants. As the impact of drought negatively affects the performance of both seeds and seedlings, water stress is the main environmental factor that limits its naturalization in open scrub vegetation, which covers large areas of sandy coastal plains in many parts of the world (Araújo and Pereira 2002). The different formations of the restingas along the Brazilian coast have different percentage of cover and variation in the water availability (Lacerda *et al.* 1993). As high light conditions and high water availability increase its seed germination and young plants survival, *C. equisetifolia* naturalization may be favoured in the formations of the restingas that has mainly open areas near water bodies.

### Persistent soil seed bank may favour invasion

Seed longevity under both storage (buried in the soil and cold/dry laboratory) conditions over 24 months was similar. These results, together with the small seed mass and the low moisture content at maturity, suggest that its seeds exhibit long-lived (orthodox) storage behaviour. The capacity to form a persistent soil seed bank for potentially up to 50 months are likely due to the dry climate and low rainfall in the restinga of State Park of Costa do Sol (Barbiére 1984); these conditions inhibit seed deterioration, soil microbial activity and decomposition processes (Cuneo *et al.* 2010). As *C. equisetifolia* seeds can remain viable in the soil for almost 4 years, they may germinate whenever environmental conditions are favourable for germination (Baskin and Baskin 2014). All these features increase the overall probability of recruitment and further naturalization of this species on sandy coastal plains of Brazil.

### Seeds can germinate across a broad range of temperatures and light conditions

*Casuarina equisetifolia* seeds had a fast physiological response when in contact with water, and germination started in 3–4 days after water uptake in optimal germination temperatures (30 and 35 °C). For small-seeded species, high germination rate is crucial for the recruitment of new individuals, mainly in environments with water restrictions, as is the case in the restingas (Martins *et al.* 2012). The capacity to germinate under a wide range of temperature conditions, including low (15 °C) and high alternating temperatures (40/20 °C), although with decrease in the germination rate, is also an important factor for a population to become naturalized in the restinga, where the temperatures can range from 21 to 31 °C (mean of 25 °C) inside the patches, and from 19 to 44 °C (mean of 30 °C) in open areas (Matos 2014). Nonetheless, the bare sand of the restinga may reach temperatures as high as 70 °C at the peak of radiation during mid-summer, in which the recruitment via seeds is restricted to a few species (Scarano 2002). In relation to the light conditions, small seeds of some species often require light for germination (Milberg *et al.* 2000), however, *C. equisetifolia* seeds are negatively photoblastic, and darkness only partially prevents its germination, although it depends on the interaction of the light with the temperature (Baskin and Baskin 2014).

The high germination percentage of *C. equisetifolia* seeds across a wide range of temperature and light conditions was evidence of its robustness (i.e. the constant expression of a particular phenotype despite genotypic and environmental variation; Waddington 1942). This increases its capacity to become naturalized in a high heterogeneous environment of temperature and light conditions, such as the restinga (Scarano 2002; Matos 2014). In addition, germination rate increased in response to favourable conditions of temperature and water availability, indicating that this species displays germination plasticity. A potential advantage of germination plasticity is the opportunistic germination response to favourable environmental conditions (Richards *et al.* 2006). Germination plasticity may have adaptive value if it enables a species to establish in variable environments where resource levels ﬂuctuate (Wainwright and Cleland 2013), as occurs in the restingas (Matos 2014). Both robustness of germination to a range of conditions and plastic fitness response to the environment may enhance the ability of alien species to invade new ecosystems (Richards *et al.* 2006; Wainwright and Cleland 2013).

### Salinity and drought reduce seed germination

Salinity and drought tolerance are also two important environmental determinants for plant recruitment on sandy coastal plains (Martins *et al.* 2012; Lai *et al.* 2015). Although *C. equisetifolia* colonizes extensive sandy areas (Morton 1980), its germinability (percentage and rate) was very sensitive to both salt and water stresses. Germination sensitivity to salt stress has been reported previously for this species (Tani and Sasakawa 2003) and for other 10 *Casuarina* species (Clemens *et al.* 1983). The germination pattern of this species is typical of halophyte species (*sensu* Woodell 1985), where seeds retain viability under saline soils and germinate in favourable conditions (e.g. after a rainy period, when the salt is leached from the substrate). In addition to halophyte seed behaviour, *C. equisetifolia* seedlings show salt stress tolerance related to physiological and biochemical mechanisms (Clemens *et al.* 1983; Tani and Sasakawa 2003). Therefore, the halophyte behaviour allows *C. equisetifolia* seeds to become quiescent in response to salt–water stresses and ensure a fast and high germination when these limiting factors are overcome. This may be another important adaptive strategy for *C. equisetifolia* to become naturalized in the restingas.

### Drought and shade reduce survival and growth of young plants

Young plants showed lower tolerance to shade and water stress than seed germination. Although its seeds have the capacity to germinate in environments with low levels of light, young plants are shade-intolerant and will not survive. Thus, even if *C. equisetifolia* seeds germinate inside vegetation patches, seedlings will not establish (T.G. Zimmermann *et al.* unpubl. data). In areas with high availability of water, young plants of *C. equisetifolia* can survive in a broad range of light conditions, except under deep shade (< 2 % of light), a condition that is often found inside vegetation patches (Matos 2014). Mainly in the restinga, tolerance of high light intensities may enhance plant survival. As for germination, water availability is crucial for the survival of young plants of *C. equisetifolia*. This species can tolerate dry climates only if the roots can grow down to the water table (Whistler and Elevitch 2006). Therefore, this tree has the capacity to become naturalized mainly in areas adjacent to watercourses. As in *C. equisetifolia*, distance to water bodies was also one of the main determinants of naturalization of *C. cunninghamiana* in South Africa (Potgieter *et al.* 2014*b*).

In contrast to *C. equisetifolia*, shaded microsites beneath the canopy in vegetation patches is the most favourable niche for regeneration for many restinga species (Matos 2014). As ﬂuctuation in resource availability is a key factor controlling invasibility (Davis *et al.* 2000), alien species will be more successful at invading communities if they do not encounter intense competition from resident species for available resources such as light. Therefore, following a disturbance, a light increment followed by a rainy event will increase the susceptibility of the restinga to the invasion of *C. equisetifolia.*

*Casuarina equisetifolia* showed differences in growth rate and biomass allocation in response to changes in light intensity. Although plant survival was high at 15 % light levels under high water conditions, shading decreased growth and the young plants exhibited shade avoidance responses, such as high shoot: root ratio, slenderness index, stem mass fraction and specific stem length (Ryser and Eek 2000). Under high water, *C. equisetifolia* exhibits similar growth between conditions of 100 % of light and in the *Casuarina* stand (70 % of light), which improves its potential to become naturalized in open areas. In attempt to minimize evaporative demand (Bloor and Grubb 2004), *C. equisetifolia* showed changes in leaf morphological traits under high light conditions, which results in lower specific leaf area and leaf area ratio. This adaptation is important for an alien species to become naturalized in habitats with low water availability, such as the restinga. In addition, specific leaf area is a plant trait that has shown to be associated with invasive success across a broad range of species (van Kleunen *et al.* 2010; Leishman *et al.* 2014).

### Low phenotypic plasticity and high phenotypic integration of traits

Although *C. equisetifolia* showed germination plasticity, young plants exhibited low morphological plasticity in response to light. Low phenotypic plasticity has also been reported in other invasive species in habitats with multiple stress factors, such as in *Acacia longifolia* in Mediterranean dunes (Peperkorn *et al.* 2005), indicating that morphological plasticity may be advantageous in favourable environments, whereas stability is more beneficial under adverse conditions (e.g. Valladares *et al.* 2000, 2007).

Several studies have shown that phenotypic integration tends to increase with environmental stress, and the higher levels of integration observed in these habitats should constrain the plastic responses of plants (Gianoli 2004; García-Verdugo *et al.* 2009; Gianoli and Palacio-López 2009). Nevertheless, in the stressful environment (shade) occurred a positive effect of phenotypic integration on the plastic expression of *C. equisetifolia* morphological traits. As long as environmental conditions ameliorate it is likely that this alien species does not need to coordinate the phenotype to exhibit plasticity. Therefore, phenotypic integration may not constrain phenotypic plasticity of plants in adverse conditions. The values of phenotypic integration index for *C. equisetifolia* was similar between shady (2.20) and sunny (2.78) conditions, even though the magnitude of individual correlations often changed from one environment to another. These values may be considered high, since studies showed that ranges from 0.77 to 1.63 (Waitt and Levin 1993; Boucher *et al.* 2013). A high degree of phenotypic integration may thus be a facilitator of adaptation, because it can reduce maladaptive variation (Armbruster *et al.* 2014), which is an important factor in the evolutionary ecology of this species. This appears to be an important strategy for an alien species to become naturalized in environments with multiple stress conditions. Nonetheless, the role of phenotypic integration in invasiveness remains poorly understood (Godoy *et al.* 2012), and more work is needed to elucidate the function of the trait correlations along the naturalization–invasion continuum.

The large production of small seeds (Apfelbaum *et al.* 1983), associated with anemochory and hydrochory dispersal syndromes (Morton 1980, Rentería 2007, Wheeler *et al.* 2011), the long-term persistence of seeds in the soil, high germination, survival and growth under high light, higher efficiency in allocating biomass on structures for water absorption (low shoot: root ratio) and light-capturing (high leaf mass fraction), together with the low phenotypic plasticity and high phenotypic integration, are crucial factors that allow *C. equisetifolia* to overcome barriers to reproduction and survival and to become naturalized on sandy coastal plains. These traits, coupled with the salt tolerance and symbiotic associations (Zhong *et al.* 1995, Diagne *et al.* 2013) enable this species to invade mainly open, sandy habitat, adjacent to watercourses, especially along coastlines, where disturbances have occurred.

### Management strategies

To limit further naturalization of *C. equisetifolia* and to prevent it from becoming invasive in the restingas planting of the species should be avoided, especially in open areas near water bodies. Removal of *C. equisetifolia* is difficult, because of its capacity for vigorous regrowth (Morton 1980), and seeds can remain viable in the soil for almost 4 years. Thus, we recommend the periodic removal of cones and seeds especially at the edge of the *Casuarina* stands, to prevent recruitment and further invasion in the restinga. As *C. equisetifolia* does not tolerate shade and drought and invades mainly degraded areas, one of the best ways of hampering its naturalization in the restinga is to conserve the remaining fragments. Therefore, habitat disturbance should be minimized to reduce opportunities for the colonization of this species. Where habitats are disturbed, immediate replanting with native vegetation is required. Nevertheless, restingas have been severely threatened mainly by anthropogenic disturbances which altering the key processes that naturally make restingas resistant to *C. equisetifolia* invasion. Further degradation is sure to lead to the status of this species changing from naturalized to invasive in large areas in Brazil.

## Conclusions

The long-term persistence of seeds in the soil, the capacity to germinate across a wide range of temperature and light conditions and the high survival rate of the young plants in conditions with moderate and high irradiance with high soil moisture are key factors that favour the naturalization of *C. equisetifolia.* Thus, areas in the restingas and on sandy coastal plains that present high-light conditions and are near water bodies are prone to naturalization of the introduced population of this species. As young plants showed lower tolerance to shade and water stress than seed germination, even if the seeds can germinate, young plants will not survive under low light (e.g. vegetation patches). Although this species exhibited high germination plasticity, young plants showed low phenotypic plasticity, which is important in habitats with multiple stress factors (Valladares *et al.* 2000, 2007). The high phenotypic integration is an important factor in the evolutionary ecology of this species because can facilitate adaptation, thereby improving the chances of this species becoming naturalized in environments with harsh conditions. As *C. equisetifolia* does not tolerate shade and drought and invades mainly degraded areas, conservation of the restingas is crucial to limit invasion of this species.

## Sources of Funding

This study was supported by the Rio de Janeiro Botanic Garden Research Institute (JBRJ), Coordenação de Aperfeiçoamento de Pessoal de Nível Superior (CAPES) and Fundação de Amparo a Pesquisa do Estado do Rio de Janeiro (FAPERJ).

## Contributions by the Authors

T.G.Z. and A.C.S.A. conceived the idea. T.G.Z. conducted the experiments and ran the statistics. T.G.Z and A.C.S.A. led the writing with assistance of D.M.R.

## Conflict of Interest Statement

None declared.

## Supplementary Material

Supplementary Data

## Supporting Information

The following additional information is available in the online version of this article —

**Figure S1**. Patchy structure of the restinga (patchy shrub vegetation).

**Table S2.** *F*-test of significance for main effects and interactions in an analysis of covariance (ANCOVA) for the effects of storage and light conditions in germination of *Casuarina equisetifolia* seeds.

**Figure S3.** Survival curves of young plants of *Casuarina equisetifolia* in response to light (A), water stress (B), drought under high light (C) and drought under low light (D).

**Table S4.** Spearman’s rank correlation matrices among 12 morphological traits of young plants of *Casuarina equisetifolia*.
