# Supplementary material for: Experimental assessment of factors mediating the naturalization of a globally invasive tree on sandy coastal plains: a case study from Brazil
Source: AoB Plants. 2016 Aug 2;8:plw042. doi: 10.1093/aobpla/plw042 (PMC4975072; doi:10.1093/aobpla/plw042)
Supplement: Supplementary Data [file supp_8_plw042_index.html]

Experimental assessment of factors mediating the naturalization of a globally invasive tree on sandy coastal plains: a case study from Brazil — Supplementary Data 

# Experimental assessment of factors mediating the naturalization of a globally invasive tree on sandy coastal plains: a case study from Brazil

## Supplementary Data

files

- Supplementary Data - zip file
