# Supplementary material for: Experimental assessment of factors mediating the naturalization of a globally invasive tree on sandy coastal plains: a case study from Brazil
Source: AoB Plants. 2016 Aug 2;8:plw042. doi: 10.1093/aobpla/plw042 (PMC4975072; doi:10.1093/aobpla/plw042)
Supplement: Supplementary Data [file supp_plw042_suppl_data.zip › aobplants-15324-s06.docx]

# OPEN ACCESS – RESEARCH ARTICLE

**Experimental assessment of factors mediating the naturalisation of a globally invasive tree on sandy coastal plains: a case study from Brazil**

**Thalita G. Zimmermann^1,*^, Antonio C. S. Andrade^1^, David M. Richardson^2^**

###

^1^ Laboratório de Sementes. Instituto de Pesquisas Jardim Botânico do Rio de Janeiro. Rua Pacheco Leão, 915, Jardim Botânico, 22460-030, Rio de Janeiro, RJ, Brasil.

^2^ Centre for Invasion Biology, Department of Botany and Zoology, Stellenbosch University, Matieland, 7602, South Africa.

*Corresponding author

Corresponding author’s e-mail address: [thalitagabriella@gmail.com](mailto:thalitagabriella@gmail.com)

**Factors mediating the naturalisation of a globally invasive tree**
