# Supplementary material for: Experimental assessment of factors mediating the naturalization of a globally invasive tree on sandy coastal plains: a case study from Brazil
Source: AoB Plants. 2016 Aug 2;8:plw042. doi: 10.1093/aobpla/plw042 (PMC4975072; doi:10.1093/aobpla/plw042)
Supplement: Supplementary Data [file supp_plw042_suppl_data.zip › aobplants-15324-s01.docx]

**
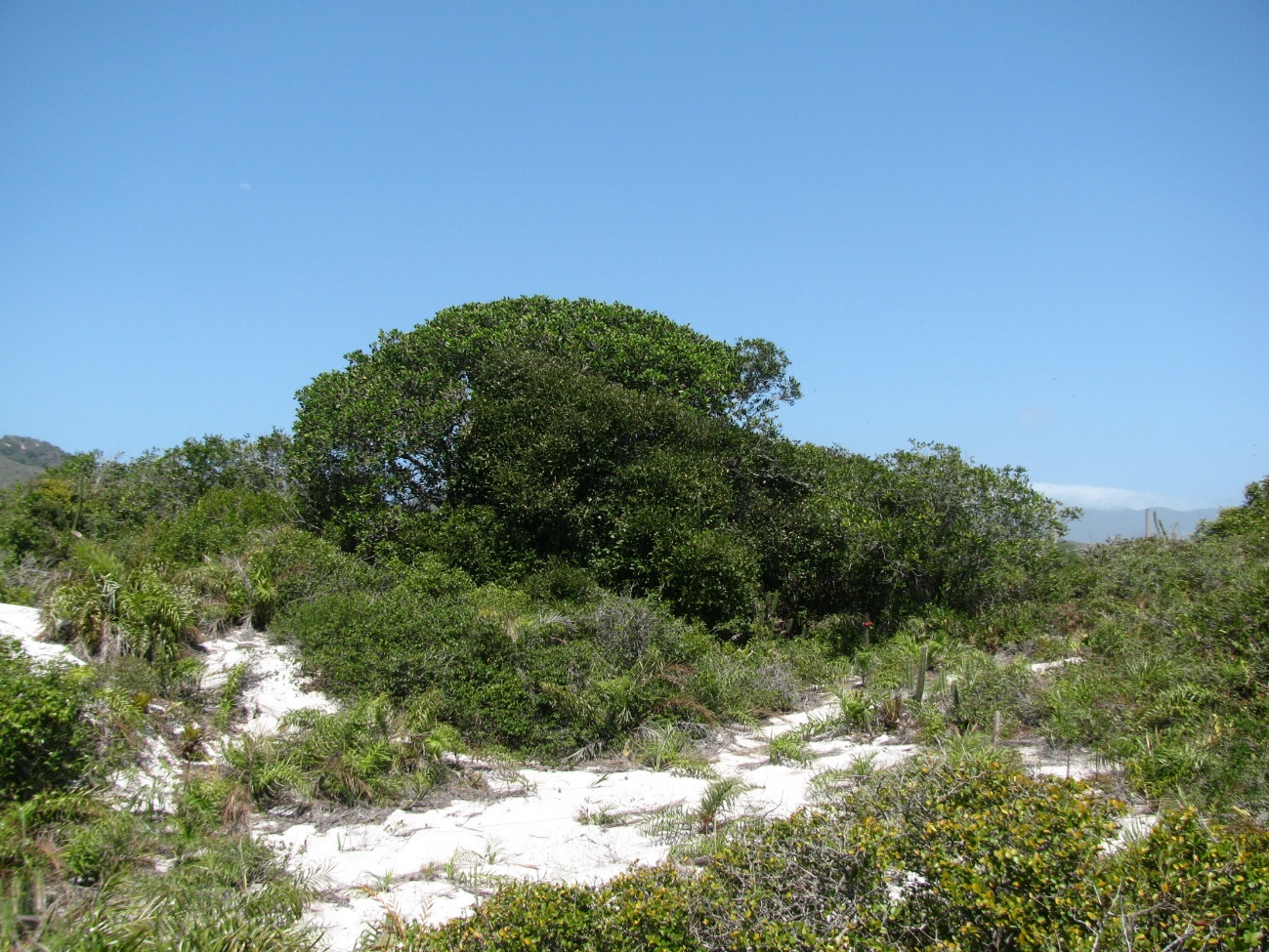
**

**File 1.** Figure. Patchy structure of the restinga (open scrub vegetation) with two distinct microsites: vegetation patches that support a high diversity and abundance of species; and open areas around the patches, usually covered by a low diversity of herbaceous vegetation.
