# Supplementary material for: Experimental assessment of factors mediating the naturalization of a globally invasive tree on sandy coastal plains: a case study from Brazil
Source: AoB Plants. 2016 Aug 2;8:plw042. doi: 10.1093/aobpla/plw042 (PMC4975072; doi:10.1093/aobpla/plw042)
Supplement: Supplementary Data [file supp_plw042_suppl_data.zip › aobplants-15324-s02.docx]

**File 2.** Table. F-test of significance for main effects and interactions in an Analysis of covariance (ANCOVA) for the effects of storage (cold storage (-18**°**C) vs. soil storage (buried in the soil)) and light conditions (light vs. dark) in germination percentage (GP) and rate (GR) of *Casuarina equisetifolia* seeds at intervals of 1, 3, 6, 9, 12, 18 and 24 months of storage.

| **Effect** | **DF** | **GP GR** | | | |
| --- | --- | --- | --- | --- | --- |
|  |  | ***F*** | ***p*** | ***F*** | ***P*** |
| *Cold storage vs. Soil storage* |  |  |  |  |  |
| Storage conditions | 1 | 1.980 | .163 | 104.347 | .000 |
| Storage time | 1 | 153.640 | .000 | .008 | .931 |
| Storage conditions vs. Storage time | 1 | 1.180 | .281 | 90.190 | .000 |
| *Soil storage light vs. Soil storage dark* |  |  |  |  |  |
| Light conditions | 1 | 25.617 | .000 | 55.079 | .000 |
| Storage time | 1 | 81.933 | .000 | .196 | .660 |
| Light conditions vs. Storage time | 1 | 6.721 | .012 | 2.891 | .095 |
