# Supplementary material for: Experimental assessment of factors mediating the naturalization of a globally invasive tree on sandy coastal plains: a case study from Brazil
Source: AoB Plants. 2016 Aug 2;8:plw042. doi: 10.1093/aobpla/plw042 (PMC4975072; doi:10.1093/aobpla/plw042)
Supplement: Supplementary Data [file supp_plw042_suppl_data.zip › aobplants-15324-s03.docx]

**C**

**B**

**A**

**D**

**File 3.** Figure. Survival curves of young plants of *Casuarina equisetifolia* (n=15) in response to light (A), water stress (B), drought under high light (70% and 100% of light; C); drought under low light (2 and 15% of light; D). Survival analysis was performed with the Kaplan-Meier product limit method. The letter codes indicate homogeneous groups (Log-rank test).
