# Supplementary material for: Experimental assessment of factors mediating the naturalization of a globally invasive tree on sandy coastal plains: a case study from Brazil
Source: AoB Plants. 2016 Aug 2;8:plw042. doi: 10.1093/aobpla/plw042 (PMC4975072; doi:10.1093/aobpla/plw042)
Supplement: Supplementary Data [file supp_plw042_suppl_data.zip › aobplants-15324-s04.docx]

**File 4.** Table. Spearman’s rank correlation matrices among 12 morphological traits of young plants of *Casuarina equisetifolia* (total dry mass – TDM; root: shoot ratio – RS; slenderness index – SI; leaf mass fraction – LMF; stem mass fraction – SMF; root mass fraction – RMF; specific stem length – SSL; specific root length – SRL; total leaf area – TLA; total leaf mass – TLM; specific leaf area – SLA and leaf area ratio – LAR). Matrix shows correlations coefficients for traits measured at 15% (A), 70% (B), and 100% (C). Significant correlations at p< 0.05 are show in bold.

| (A) | TDM | SR | SI | LMF | SMF | RMF | SSL | SRL | TLA | TLM | SLA | LAR |
| --- | --- | --- | --- | --- | --- | --- | --- | --- | --- | --- | --- | --- |
| TDM | 1.00 |  |  |  |  |  |  |  |  |  |  |  |
| SR | -0.13 | 1.00 |  |  |  |  |  |  |  |  |  |  |
| SI | -0.52 | 0.03 | 1.00 |  |  |  |  |  |  |  |  |  |
| LMF | 0.12 | 0.47 | 0.12 | 1.00 |  |  |  |  |  |  |  |  |
| SMF | -0.13 | **0.73** | -0.07 | -0.16 | 1.00 |  |  |  |  |  |  |  |
| RMF | 0.08 | **-0.81** | -0.15 | **-0.82** | -0.30 | 1.00 |  |  |  |  |  |  |
| SSL | **-0.93** | 0.02 | **0.70** | 0.03 | -0.07 | -0.13 | 1.00 |  |  |  |  |  |
| SRL | **-0.80** | 0.31 | **0.56** | -0.11 | 0.34 | -0.24 | **0.79** | 1.00 |  |  |  |  |
| TLA | **0.88** | -0.20 | -0.40 | 0.38 | -0.43 | -0.04 | **-0.76** | **-0.81** | 1.00 |  |  |  |
| TLM | **0.97** | -0.10 | -0.52 | 0.25 | -0.24 | -0.02 | **-0.90** | **-0.83** | **0.96** | 1.00 |  |  |
| SLA | -0.15 | -0.42 | 0.27 | 0.33 | **-0.67** | 0.03 | 0.26 | -0.11 | 0.27 | 0.01 | 1.00 |  |
| LAR | -0.24 | -0.30 | 0.40 | 0.48 | **-0.64** | -0.13 | 0.40 | -0.04 | 0.20 | -0.08 | **0.96** | 1.00 |

| (B) | TDM | SR | SI | LMF | SMF | RMF | SSL | SRL | TLA | TLM | SLA | LAR |
| --- | --- | --- | --- | --- | --- | --- | --- | --- | --- | --- | --- | --- |
| TDM | 1.00 |  |  |  |  |  |  |  |  |  |  |  |
| SR | 0.05 | 1.00 |  |  |  |  |  |  |  |  |  |  |
| SI | 0.26 | 0.31 | 1.00 |  |  |  |  |  |  |  |  |  |
| LMF | -0.45 | 0.43 | 0.06 | 1.00 |  |  |  |  |  |  |  |  |
| SMF | 0.14 | **0.90** | 0.18 | 0.10 | 1.00 |  |  |  |  |  |  |  |
| RMF | 0.16 | **-0.85** | -0.18 | **-0.79** | **-0.60** | 1.00 |  |  |  |  |  |  |
| SSL | **-0.79** | -0.29 | 0.09 | 0.42 | -0.47 | -0.03 | 1.00 |  |  |  |  |  |
| SRL | **-0.70** | 0.47 | -0.05 | 0.52 | 0.40 | **-0.60** | 0.41 | 1.00 |  |  |  |  |
| TLA | 0.28 | 0.19 | 0.41 | 0.40 | 0.04 | -0.30 | -0.10 | -0.08 | 1.00 |  |  |  |
| TLM | **0.92** | 0.19 | 0.36 | -0.17 | 0.21 | -0.07 | **-0.70** | -0.53 | 0.38 | 1.00 |  |  |
| SLA | -0.47 | -0.12 | 0.05 | 0.40 | -0.20 | -0.15 | 0.53 | 0.48 | 0.54 | -0.37 | 1.00 |  |
| LAR | -0.53 | -0.06 | 0.10 | 0.51 | -0.15 | -0.23 | **0.60** | 0.54 | 0.51 | -0.37 | **0.97** | 1.00 |

| (C) | TDM | SR | SI | LMF | SMF | RMF | SSL | SRL | TLA | TLM | SLA | LAR |
| --- | --- | --- | --- | --- | --- | --- | --- | --- | --- | --- | --- | --- |
| TDM | 1.00 |  |  |  |  |  |  |  |  |  |  |  |
| SR | 0.07 | 1.00 |  |  |  |  |  |  |  |  |  |  |
| SI | -0.24 | 0.10 | 1.00 |  |  |  |  |  |  |  |  |  |
| LMF | 0.08 | **0.76** | 0.03 | 1.00 |  |  |  |  |  |  |  |  |
| SMF | 0.12 | **0.97** | 0.05 | **0.70** | 1.00 |  |  |  |  |  |  |  |
| RMF | -0.10 | **-0.96** | -0.06 | **-0.90** | **-0.91** | 1.00 |  |  |  |  |  |  |
| SSL | **-0.80** | -0.01 | 0.50 | 0.23 | -0.09 | -0.06 | 1.00 |  |  |  |  |  |
| SRL | **-0.64** | 0.38 | 0.24 | 0.43 | 0.30 | -0.42 | **0.69** | 1.00 |  |  |  |  |
| TLA | 0.45 | 0.43 | -0.27 | 0.47 | 0.37 | -0.46 | -0.38 | 0.13 | 1.00 |  |  |  |
| TLM | **0.88** | 0.37 | -0.17 | 0.48 | 0.40 | -0.45 | -0.55 | -0.38 | **0.61** | 1.00 |  |  |
| SLA | -0.32 | 0.12 | -0.24 | -0.07 | 0.03 | 0.01 | 0.09 | 0.35 | 0.42 | -0.29 | 1.00 |  |
| LAR | -0.30 | 0.44 | -0.14 | 0.48 | 0.32 | -0.43 | 0.27 | **0.55** | **0.66** | -0.02 | **0.80** | 1.00 |
